# All‐Inorganic Perovskite Quantum Dot‐Monolayer MoS_2_ Mixed‐Dimensional van der Waals Heterostructure for Ultrasensitive Photodetector

**DOI:** 10.1002/advs.201801219

**Published:** 2018-09-21

**Authors:** Hualin Wu, Haonan Si, Zihan Zhang, Zhuo Kang, Pingwei Wu, Lixin Zhou, Suicai Zhang, Zheng Zhang, Qingliang Liao, Yue Zhang

**Affiliations:** ^1^ State Key Laboratory for Advanced Metals and Materials School of Materials Science and Engineering University of Science and Technology Beijing Beijing 100083 P. R. China; ^2^ Beijing Municipal Key Laboratory of New Energy Materials and Technologies University of Science and Technology Beijing Beijing 100083 P. R. China

**Keywords:** mixed‐dimensional van der Waals heterostructures, MoS_2_, perovskite quantum dots, photodetectors, photogating effect

## Abstract

2D transition metal dichalcogenide (2D‐TMD) materials and their van der Waals heterostructures (vdWHs) have inspired worldwide efforts in the fields of electronics and optoelectronics. However, photodetectors based on 2D/2D vdWHs suffer from performance limitations due to the weak optical absorption of their atomically thin nature. In this work, taking advantage of an excellent light absorption coefficient, low‐temperature solution‐processability, and long charge carrier diffusion length, all‐inorganic halides perovskite CsPbI_3−_ *_x_*Br*_x_* quantum dots are integrated with monolayer MoS_2_ for high‐performance and low‐cost photodetectors. A favorable energy band alignment facilitating interfacial photocarrier separation and efficient carrier injection into the MoS_2_ layer inside the 0D–2D mixed‐dimensional vdWHs are confirmed by a series of optical characterizations. Owing to the synergistic effect of the photogating mechanism and the modulation of Schottky barriers, the corresponding phototransistor exhibits a high photoresponsivity of 7.7 × 10^4^ A W^−1^, a specific detectivity of ≈5.6 × 10^11^ Jones, and an external quantum efficiency exceeding 10^7^%. The demonstration of such 0D–2D mixed‐dimensional heterostructures proposed here would open up a wide realm of opportunities for designing low‐cost, flexible transparent, and high‐performance optoelectronics.

## 1 Introduction

2D transition metal dichalcogenide (2D‐TMD) materials have inspired worldwide efforts in fields of electronics and optoelectronics,^1^ such as logic devices,^2^ light‐emitters,^3^ photovoltaics,^4^ and photodetectors,^5^ due to their unique characteristics of favorable bandgap, high carrier transport mobility, outstanding flexibility, stability, etc. Considerable efforts have been devoted to the recently proposed staked 2D–2D van der Waals heterostructures (vdWHs) with the rapid development of 2D materials. However, devices based on such 2D–2D vdWHs suffer from performance limitations due to the strong interlayer coupling^6^ and limited modulation of the electronic structure for the two given 2D layered materials. In particular, the total optical absorption level and spectral selectivity are limited by their atomically thin nature and availability of the two layered materials, which is crucial to photocarrier generation efficacy. For example, the intrinsic photoresponse in black phosphorus‐monolayer MoS_2_ heterostructure was 0.41 A W^−1^,^7^ and 2.3 A W^−1^ in MoS_2_/WS_2_ vertical heterojunction.^8^

To tackle these problems, several strategies such as integrating with strong light absorption materials,^9^ surface interface engineering,^10^ and novel architectures design,^11^ etc., have been proposed. Mixed‐dimensional vdWHs (MvdWHs),^12^ which electronically couple 2D‐layered materials with materials of different dimensions such as 0D quantum dots (QDs),^13^ 1D nanowires,^14^ and 3D films,^9^, ^15^ have been considered to be a promising device architecture. Organic–inorganic halide perovskites, considered as the next‐generation solar‐cell materials,^16^ have been integrated with MoS_2_ for high performance photodetector due to their excellent light absorption coefficient. Great breakthrough has been made in this structure, of which the photoresponsivity was strikingly enhanced by a factor of 7.7 compared with that of pristine MoS_2_ device.^9^ Despite such enhancement, the relatively thicker of perovskite layer lengthened the electron transport path and increased the trap‐mediated nonradiative recombination which led to a long‐response time (6.17 s).

Semiconducting 0D QDs with superior high‐absorption coefficient, large spectral coverage, tunability bandgaps, and low‐cost solution processing were also considered as an ideal sensitizer to combining with other light‐sensitive materials for improving photoresponse.^17^ Benefiting from the greater generation as well as efficient and fast extraction of photoexcited carriers, devices based on such 0D–2D MvdWHs have been demonstrated for ultrasensitive and broadband detection with high efficiency and photogain.[[qv: 13a,b]] For example, the photoresponsivity of PbS QDs/MoS_2_ MvdWHs was reported to exceed 10^5^ A W^−1^,[[qv: 13b]] and HgTe QDs/MoS_2_ structure had a broadband photodetection beyond 2 µm.^18^ Although various of QDs integrating with 2D‐TMDCs have been explored, organic–inorganic perovskite QDs based 0D–2D photodector remained little investigated, in spite of its significant advantages such as low‐temperature solution‐processing, long charge carrier diffusion length, tunable bandgap, high quantum efficiency, etc.[[qv: 17a,19]]

In this work, all‐inorganic cesium lead halides perovskite CsPbI_3−_ *_x_*Br*_x_* QDs (PQDs) was integrated with monolayer MoS_2_ for low‐cost and high performance 0D–2D mixed‐dimensional heterostructured photodetector. A favorable band alignment and efficient carrier extraction by 2D‐MoS_2_ layer in this 0D–2D MvdWH was demonstrated, which facilitated to the photocarrier generation efficacy and photogating effect. Owing to the synergistic effect of photogating effect and the modulation of Schottky barriers, the optimized device exhibited an extremely high photoresponsivity of 7.7 × 10^4^ A W^−1^, a specific detectivity of ≈5.6 × 10^11^ Jones, and an ultrahigh external quantum efficiency exceeding 10^7^%. The demonstration of such 0D–2D mixed‐dimensional structure here would open up a wide opportunity for designing flexible, transparent, and high‐performance optoelectronics.

## 2 Results and Discussion

**Figure 1:**
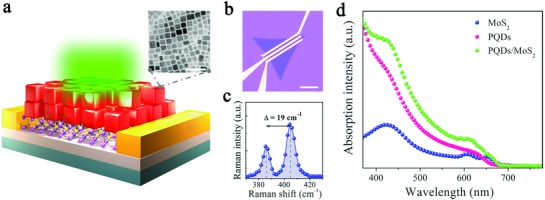
Schematic model of the device and optical characterization of the PQDs/MoS_2_ MvdWH. a) Schematic model of the device with its inset shows a typical TEM image of the PQDs. The scale bar is 50 nm. b) Optical image of the as‐fabricated device. The scale bar is 10 µm. c) Raman spectra of pristine MoS_2_ monolayer. d) UV–vis absorption of the pristine MoS_2_, pure PQDs, and the MvdWH.

The device schematic model of the CsPbI_3−_ *_x_*Br*_x_* PQDs/MoS_2_ monolayer mixed‐dimensional phototransistor was shown in **Figure** **1**a. The monolayer MoS_2_, which served as the channel of the hybrid phototransistor, was synthesized by oxygen‐assisted chemical vapor deposition^20^ and transferred onto SiO_2_/Si substrate by poly(methyl methacrylate) (PMMA)‐assisted transfer method. Then the as‐synthesized colloidal CsPbI_3−_ *_x_*Br*_x_* QDs dilute solution was uniformly spin‐coated onto the MoS_2_ monolayer. Before spin‐coating, the density of ligands in PQDs was controlled to improve the conductance by the method reported by Li et al.^21^ The transmission electron microscope (TEM) image shows that the PQDs had a typical cubic shape with an average diameter of 15 nm (inset of Figure 1a). The channel was defined by standard photolithography and electron‐beam lithography (2 µm in length and 20 µm in width, as shown in Figure 1b); then Au source/drain contacts (100 nm in thickness) were fabricated on MoS_2_ by thermal evaporation. Two characteristic Raman peaks of MoS_2_ monolayer were observed at 385.8 cm^−1^ (E^1^_2g_ mode) and 404.8 cm^−1^ (A_1g_ mode); the difference between E^1^_2g_ and A_1g_ mode peaks was 19 cm^−1^, well corresponding to the theoretical value of monolayer MoS_2_ (Figure 1c).^22^

To investigate the optical characteristics of the PQDs/MoS_2_ MvdWH, we performed UV–vis absorption spectra and photoluminescence (PL) spectra analysis on the pristine MoS_2_ monolayer, PQDs, and PQDs/MoS_2_ hybrid system. The UV–vis absorption spectrum of the pristine MoS_2_ layer presented two excitonic absorption peaks between 600 and 700 nm (Figure 1d), which were ascribed to the direct excitonic transitions at K point in the Brillouin zone.^23^ The significantly increased light absorption below 670 nm indicates that monolayer MoS_2_ possessed a direct optical band gap of 1.85 eV. For the PQDs, the bandgap was determined to be 1.91 eV from the absorption spectrum, which was also close to the near‐band‐edge emission of 1.92 eV in the PL spectra (Figure S1, Supporting Information). In comparison, the PQDs/MoS_2_ MvdWH showed enhanced absorption below 645 nm wavelengths, which resulted from the synergetic absorption effect of PQDs and MoS_2_ layer.

Since Kelvin probe force microscopy (KPFM) is sensitive to the surface work function (*W*_F_) of materials,^24^ we performed KPFM analysis to gain insight into the electronic structure of PQDs/MoS_2_ MvdWH. Dual‐pass amplitude‐modulated‐KPFM mode on Bruker Dimension Icon with a Pt/Ir‐coated probe (SCM‐PIT, *K* = 2.8 N m^−1^, Bruker) was employed to measure the surface potential at ambient atmosphere. Before measuring, the work function of the tip was calibrated by taking the contact potential difference (CPD) on Cu foil. The CPD was nearly zero which indicated that the work function of the tip was close to that of Cu (≈4.8 eV). The measured CPD between the sample and the tip can be expressed by the following equation[[qv: 24b]]: *e* × *V*_CPD_ = *W*_F, tip_ − *W*_F, sample_, where *W*_F, tip_ and *W*_F, sample_ are the work functions of the tip and sample, respectively, and *e* is the elementary charge.

**Figure 2:**
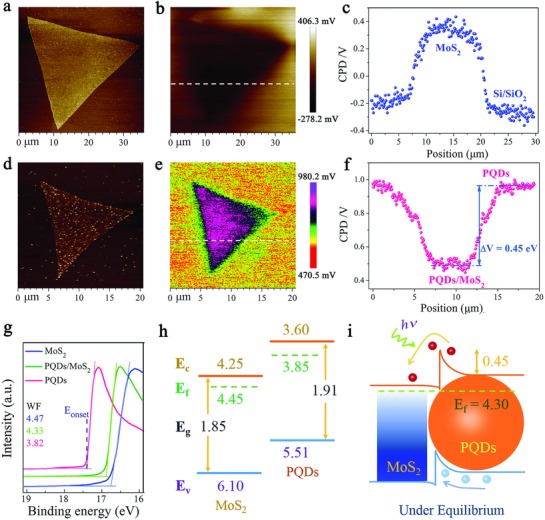
KPFM characterization and UPS experimentally determined energy level diagram of the PQDs/MoS_2_ MvdWH. a,d) Topography profile, b,e) spatial maps of the contact potential difference (CPD), and c,f) line profiles of CPD along the white dotted line marked in panels (b) and (e) for pristine MoS_2_ and PQDs/MoS_2_ MvdWH, respectively. g) The WF measured by UPS. h) Energy level diagrams for PQDs and MoS_2_ before contact and i) band diagram for PQDs/MoS_2_ heterojunction under equilibrium in dark.

The topography profile of the pristine MoS_2_ layer and PQDs/MoS_2_ MvdWH was recorded under tapping mode in **Figure** **2**a,d, respectively, and the corresponding CPD distribution images were shown in Figure 2b,e. Figure 2c,f shows the CPD line profiles along the white dotted line marked in Figure 2b,e, respectively. Assuming the *W*_F, tip_ remained constant, the local surface work function of the sample could be determined in situ through the fluctuation of CPD values. We found a decreasing trend of CPD relative to the tip from the SiO_2_/Si (≈−0.2 V in average) toward the MoS_2_ layer (0.35 V in average, blue line profile in Figure 2c). So the work functions of SiO_2_/Si and MoS_2_ layer, *W*_F, SiO2/Si_ ≈ 5.0 eV and *W*_F, MoS2_ ≈ 4.45 eV, can be calculated by the equation as aforementioned, respectively. Note that the obtained work function of the monolayer MoS_2_ is a reasonable value, since it is quite close to previously reported values.^25^ Similarly, in the case of PQDs/MoS_2_ MvdWH, there was an increasing CPD from the PQDs film (≈0.95 V in average) toward the PQDs/MoS_2_ MvdWH (≈0.5 V in average, pink line profile in Figure 2f), and the work functions of PQDs and PQDs/MoS_2_ MvdWH were 3.85 and 4.3 eV, respectively. To verify the work functions obtained from KPFM, we performed ultraviolet photoelectron spectroscopy (UPS) analysis (Figure 2g). The work functions of MoS_2_, PQDs, and PQDs/MoS_2_ are determined to be 4.47, 3.82, and 4.33 eV, respectively, which were calculated by equation *W*_F_ = *hν* − *E*_onset_ (where *hν* = 21.22 eV is the incident photon energy, and *E*_onset_ is the onset level related to the secondary electrons),[[qv: 25a,26]] coinciding with the KPFM results.

Additionally, the conduction bands of MoS_2_ monolayer and PQDs were reported to be 4.25[[qv: 25a]] and 3.6 eV^27^ in previous work in energy, respectively. Combining the optical band gaps obtained from the absorption spectra and the Fermi level determined by KPFM, the energy band structure of PQDs/MoS_2_ MvdWH was determined to form a type‐II energy band alignment (Figure 2h). Specifically, the work function of PQDs on MoS_2_ monolayer increased by 0.45 eV relative to PQDs alone. This indicates the electrical junction formed when the PQDs were brought into contact with MoS_2_ monolayer, and that electrons diffused from PQDs to MoS_2_ monolayer to align the Fermi level and reach a new equilibrium. Due to the charge transfer, the energy band bent and depletion region then formed, leading to a 0.6 eV built‐in field, i.e., the Fermi level difference of two materials, at the interface. With incident laser excitation, electron–hole pairs were mainly generated in the highly light‐absorbing PQDs layer, and then separated by the built‐in field. As a result, electrons diffused to the MoS_2_ side while holes were left in the PQDs side, as illustrated in Figure 2i.

**Figure 3:**
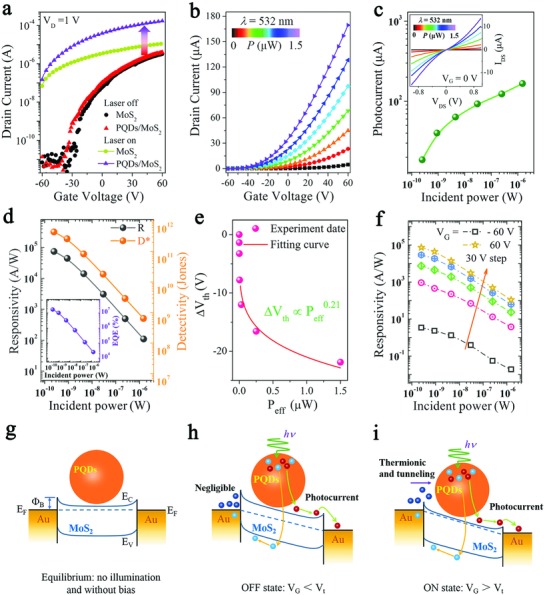
Optoelectronic performance and the schematic of channel current transport mechanism. a) Transfer characteristics for pristine MoS_2_ and PQDs/MoS_2_ hybrid phototransistors both in dark and under illumination of 532 nm at *V*_D_ = 1 V. b) Photoinduced transfer characteristics of the MvdWH based phototransistor under a range of efficient illumination powers (from dark to 1.5 µW). c) Photocurrent obtained from panel (b) at *V*_G_ = 60 V. The inset shows the *I*_D_–*V*_D_ curves as a function of illumination powers. d) Photoresponsivity and specific detectivity with an inset shows the EQE as a function of illumination powers of the MvdWH based phototransistor. e) The shift of the threshold voltage (Δ*V*_th_) from panel (b) as a function of illumination powers. f) Photoresponsivity as a function of effective illumination powers under a range of gate voltage from −60 to 60 V with a fixed step of 30 V. The schematic of channel current transport mechanism and energy band diagram of the MvdWH based phototransistor under g) equilibrium conditions, h) OFF‐state, and i) ON‐state. *E*_F_, *E*_c_, *E*_V_, and Φ_B_ are the Fermi level energy, minimum conduction band energy, maximum valence band energy, and Schottky barrier height, respectively.

To evaluate the improvement in the optoelectronic performance of the PQDs/MoS_2_ hybrid phototransistor compared to the pristine MoS_2_ device, the transfer characteristics (*I*_D_ (drain current) versus *V*_G_ (gate voltage)) were investigated both in dark and under illumination at a constant drain voltage of 1 V (**Figure** **3**a). Both pristine MoS_2_ based transistor and hybrid phototransistor exhibited a typical n‐type behavior in the dark. Under light illumination (532 nm, 1.5 µW), the drain current of the hybrid phototransistor was dramatically enhanced by 15.3 times compared with pristine MoS_2_ device (obtained at *V*_D_ = 1 V), manifesting the strong absorption characteristics of PQDs and effective separation of photoexcited carriers.

**Figure 4:**
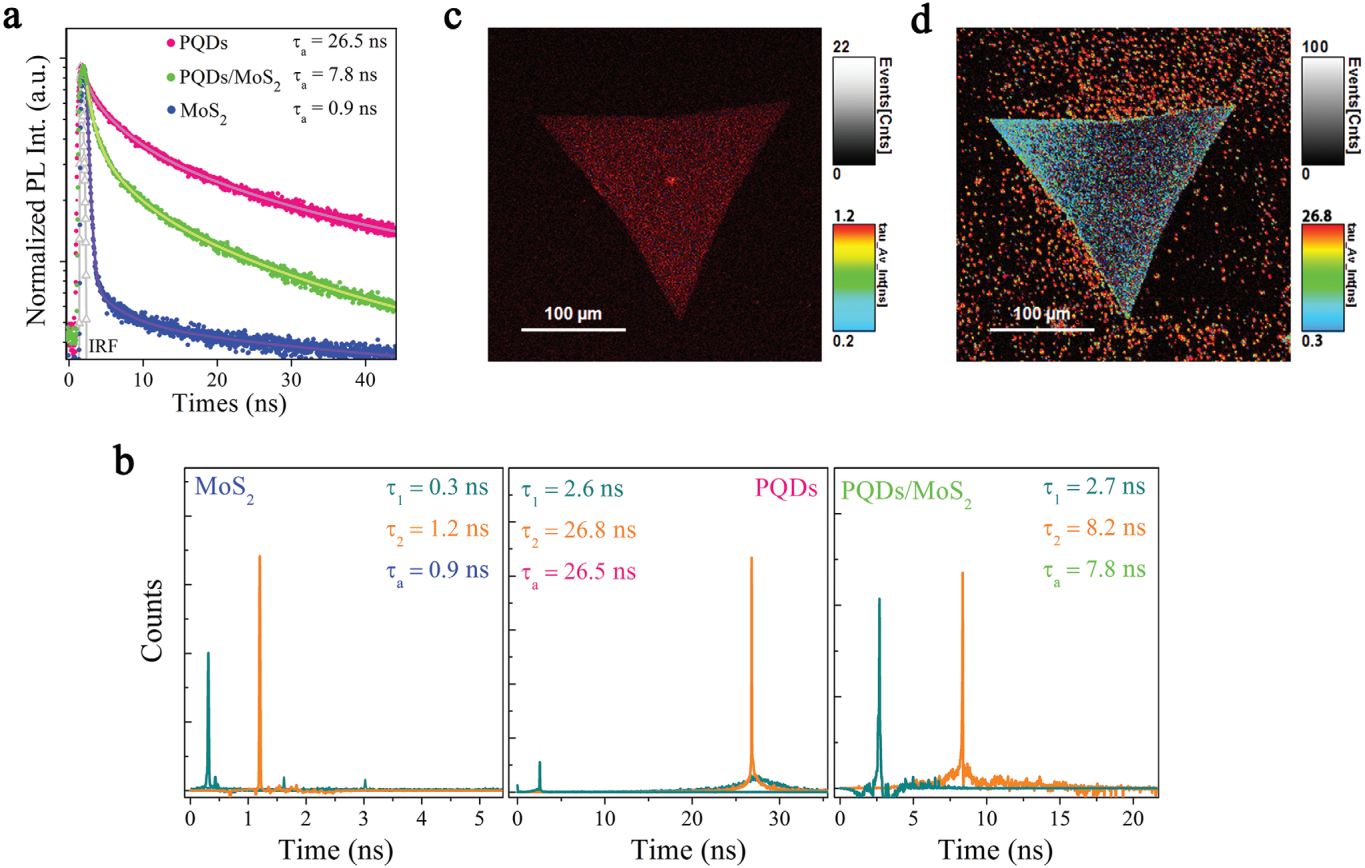
TRPL and FLIM characterizations of the PQDs/MoS_2_ MvdWH. a) TRPL measurement for pristine MoS_2_, pure PQDs, and the MvdWH. b) The distribution and proportion of the fast and slow lifetime. c,d) Fluorescence lifetime mapping for pristine MoS2 and PQDs in the MvdWH, respectively.

The photoinduced transfer curves of the PQDs/MoS_2_ mixed‐dimensional phototransistor under 532 nm illumination with a range of optical powers (from dark to 1.5 µW) are shown in Figure 3b. The curve of log‐plotted photocurrent (*I*_ph_ = *I*_light_ − *I*_dark_, obtained from **Figure** **4**b) as a function of effective illumination power (*P*_eff_) at a fixed gated voltage of 60 V is plotted in Figure 3c. As a general law, the drain current increased when the illumination power increased. The photocurrent curve could be fitted in a simple power law, *I*_ph_ ∼ *P*_eff_^α^, where the obtained exponent α via linearly fitting the curve is 0.72, indicating there existed trap‐assisted recombination at the interface of MvdWH.^28^ In addition, the *I*_D_–*V*_D_ curves (obtained at *V*_G_ = 0 V) both in dark and under illumination with different powers are shown in the inset of Figure 3c, which are symmetric and almost linearly depend on applied bias voltages, indicating the existence of small Schottky barriers at the contact interface. Moreover, the current on/off ratio over 10^4^ (Figure S2, Supporting Information) and the output characteristics under illumination and dark (Figure S3, Supporting Information) both manifest good gate modulation of the hybrid device.

Then, we analyzed two most important figures of merit for a photodetector, external photoresponsivity (*R* = *I*_ph_/*P*_eff_) and specific detectivity (D* = *RA* ^(1/2)^/(2*eI*_d_)^1/2^),^29^ where *P*_eff_ is the effective incident power (*P*_eff_ = *P*_in_ × *A*_device_/*A*_laserspot_), *A* is the effective detection area, *I*_d_ is the dark current, and *e* is the unit charge. The curves of calculated *R* and D* as functions of illumination power were plotted in Figure 3d. As is shown that the PQDs/MoS_2_ mixed‐dimensional photodetector reached a photoresponsivity (black line) of 7.7 × 10^4^ A W^−1^ and a specific detectivity (orange line) of ≈5.6 × 10^11^ Jones under illumination power in the nanowatt scale, which is quite outstanding performance compared to those previously reported TMD‐based photodetectors.[[qv: 29a,30]]

Note that both R and D* decreased exponentially with increasing incident power. This phenomenon was attributed to higher probability of scattering and recombination under stronger illumination,[[qv: 9,13b]] and higher values of *R* and D* could be obtained by lowering the illumination power. The external quantum efficiency (EQE) is defined as the number of electrons collected per incident photon bought about and expressed by the equation: $\mathrm{EQE}(\%)\;=N_{\mathrm{el}}/N_{\mathrm{ph}}\;=\frac{I_{\mathrm{ph}}}{P_{\mathrm{eff}}}\;\frac{hc}{\lambda e}\times \;100,$ 10, 31 where λ is the wavelength in nm, *h* is the Planck's constant, *c* is the speed of light in vacuum, and *e* is the elementary charge. As shown in Figure 3d, our PQDs/MoS_2_ mixed‐dimensional phototransistor yielded a very high EQE value exceeding 10^7^% at a *V*_G_ of 60 V, which is several orders of magnitude higher than those of previously reported ones.^10^, ^31^, ^32^ It could be considered that such ultrahigh EQE may primarily result from the photogating effect, a prevalent mechanism in 2D materials and their MvdWHs, arising from trapped long‐life carriers by surface and interface trap states. In order to explore the mechanisms that underlay the outstanding performance of our MvdWH, we extracted the shift of threshold voltage (Δ*V*_th_) from the relatively linear part of the transfer curves in Figure 3b, and the Δ*V*_th_ as a function of effective incident power (*P*_eff_) was plotted in Figure 3e. The curve matched well with the function $\Delta V_{\mathrm{th}}=aP_{\mathrm{eff}}^{b},$ 33 and the obtained *b* value ≈ 0.21, smaller than 1, confirmed the existence of photogating effect in our phototransistor. Specifically, the dominate photocurrent generation mechanism can be determined through a simple power‐law, *I*_ph_ ∝*P*_eff_^α^,[[qv: 33b]] where we extracted α by linear fitting the log‐plotted of *I*_ph_ versus *P*_eff_ (Figure S4, Supporting Information). The obtained α ranges from 0.83 to 0.22 as the applied gate voltage increases, manifesting that the dominate mechanism can be tuned from photoconduction to high gain photogating when the applied gate voltage gradually increased.

Moreover, as shown in Figure 3f, the photoresponsivity gradually increased with the increasing gate voltage under light with each intensity. This behavior could be explained by the influence of gate voltage on energy band structure at the contact interface, which led to two distinct regimes of channel current transport mechanisms, i.e., the depletion regime (*V*_G_ < *V*_t_, OFF state) and the accumulation regime (*V*_G_ > *V*_t_, ON state).[[qv: 29b,34]] As is illustrated in Figure 3g, without applying gate electric field, the device was in its equilibrium state, and the Schottky barrier at the interface was negligible. In the OFF state (Figure 3h), the Schottky barrier at the contact interface was remarkably increased under applied negative gate electric field. As a result, the thermionic and tunneling current became negligible, so the photogenerated current predominantly contributed to the channel current under illumination. The dark current can be strongly reduced when the device at OFF state. While in the ON state (Figure 3i), apart from photogenerated current, the thermionic and tunneling current gradually increased due to the lowered barrier height, which significantly increased the channel current. Thus, benefiting from the synergistic effect of photogating mechanism and the Schottky barriers modulation, the channel current was drastically enhanced at ON state, leading to the ultrahigh photoresponsivity.

To further investigate and verify whether the photoinduced interfacial charge transfer of our PQDs/MoS_2_ MvdWH played an important role for the photocurrent generation, we conducted time‐resolved photoluminescence (TRPL) and fluorescence lifetime imaging microscopy (FLIM) measurements. The transient photoluminescence decay curves were fitted by a two‐component exponential decay model ($F(t)=\sum a_{i}e^{-(t-t_{0})/\tau_{i}},\;i=1,2$) in Figure 4a, which was considered to consist of fast decay τ_1_ and slow decay τ_2_. The fast component related to trap‐assisted recombination at grain boundaries in pure PQDs or carrier extraction by layers in PQDs/MoS_2_ MvdWH, whereas the slow one related to the radiative recombination inside the grains or at the interface of the MvdWH.^35^

We found that the photoluminescence transients of the PQDs in the MvdWH were significantly slowed down compared with that of pure PQDs, from average 26.4 ns in pure PQDs to about 7.8 ns in the MvdWH. This phenomenon indicated the appearance of a strong quenching effect resulting from the effective charge transfer from PQDs to MoS_2_ layer.^36^ Moreover, the fast transient component proportion was drastically increased in the MvdWH compared with that of the pure PQDs, from 12.8 percent to 43.4 percent, verified the effective carrier extraction by MoS_2_ layer,^37^ as shown in Figure 4b. Furthermore, the fluorescence lifetime mapping images of the PQDs/MoS_2_ MvdWH were obtained with the samples excited by a pulsed diode laser (483 nm, 26.67 MHz) at excitation fluences of 3.5 µJ cm^−2^, filtered by 690/50 and 620/50 nm bandpass filters, and spatially mapped in Figure 4c,d, respectively. The images visually show the fluorescence lifetime of MoS_2_ layer obtained from 690/50 nm bandpass filters, which was determined to be 0.9 ns and is comparable to previously reported values^38^; while the fluorescence lifetime of PQDs on MoS_2_ layer, obtained from 620/50 nm bandpass filters, was significantly shortened (dark blue triangle) than that of pure PQDs on glass substrate (bright red dots), which are well consistent with the TRPL results in Figure 4a. Thus, under illumination and positive gate electric field, the flow of photo‐excited carriers in PQDs to MoS_2_ channel was accelerated, leading to the dramatically increased photocurrent and ultrasensitive photoresponse.

**Figure 5:**
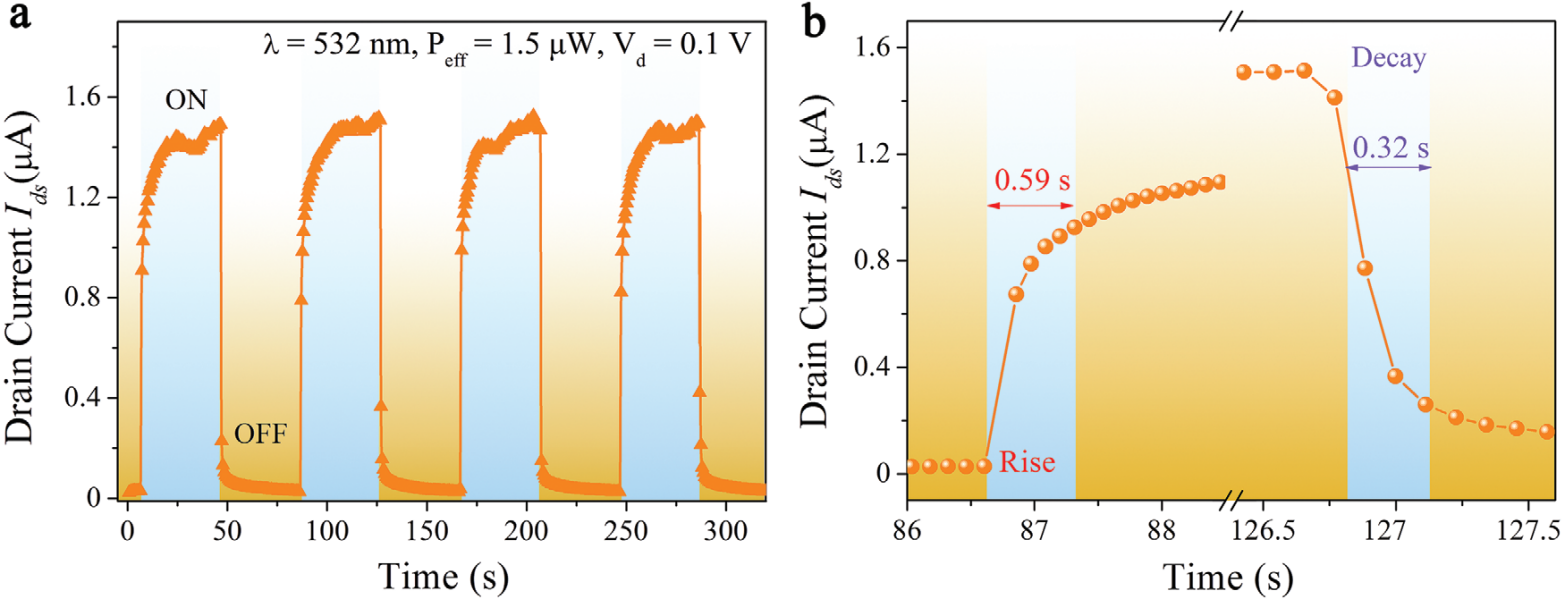
Photoswitching characteristics of the PQDs/MoS_2_ hybrid photodetector. a) Time‐dependent photoresponse under 1.5 µW pulsed illumination power over multiple cycles at *V*_D_ = 0.1 V. b) The rise and fall times in photocurrent extracted from panel (a).

Finally, the photoswitching characteristic of the PQDs/MoS_2_ hybrid photodetector was investigated under 1.5 µW pulsed illumination power over multiple cycles at *V*_D_ = 0.1 V (**Figure** **5**a) and the rising and decay time extracted from the dynamic curves was shown in Figure 5b. Our PQDs/MoS_2_ hybrid photodetector exhibited stable and reproducible on‐off photoswitching property, and the average rise and fall times were characterized to be 0.59 s and 0.32 s, respectively. Both the rise and fall curves in photocurrent can be fitted to a single exponential function.

## 3 Conclusion

In summary, we have demonstrated the high performance of a novel 0D–2D MvdWH photodetector based on all‐inorganic CsPbI_3−_ *_x_*Br*_x_* perovskite QDs and 2D‐MoS_2_ monolayer. A favorable energy band alignment facilitating interfacial photocarrier separation and efficient carrier injection into MoS_2_ layer inside the MvdWH were confirmed by a series of optical characterizations. As a result, the photocurrent was enhanced by 15.3‐fold. Owing to the synergistic effect of photogating effect together with the Schottky barriers modulated by gate voltage, the optimized device exhibited a high photoresponsivity of 7.7 × 10^4^ A W^−1^, a specific detectivity of ≈5.6 × 10^11^ Jones, and an EQE exceeding 10^7^%. Due to the solution‐processability of perovskite QDs together with the simple and low‐temperature preparation technology, our 0D–2D MvdWH can be further applied for low‐cost, flexible, and transparent photodetectors. Furthermore, because of the wide range bandgap‐tunability of perovskite QDs, the demonstration of such 0D–2D MvdWH may bring about more possibilities for designing diverse optoelectronic devices.

## 4 Experimental Section

*Preparation of MoS_2_ Monolayers*: Monolayer MoS_2_ was synthesized by oxygen‐assisted chemical vapor deposition method.^20^ A quartz boat that was filled with molybdenum oxide (MoO_3_) powder (0.01 g) and covered by a cleaned Si/SiO_2_ substrate was pushed in the center of a tubular furnace. Another ceramic boat filled with sulfur (S) powder was placed at the upstream region of the furnace at a lower temperature zone. The temperature was first raised to 300 °C and held for 30 min to preheat MoO_3_ powder, then to 850 °C/30 min to grow large monolayer MoS_2_ crystals. Meanwhile the S powder was heated to 180 °C by a heating belt, and the sulfur vapor flowed into the furnace by the carrier gas of Ar. Particularly, 2‐sccm O2 was used to decrease the nucleation density at the growing stage. Finally, the furnace was gradually cooled down to room temperature. During the whole process, the ultrahigh‐purity Ar was held at 500 sccm under atmospheric pressure.

*Synthesis of CsPbBr_3−x_I_x_ Quantum dots*: Colloidal CsPbI_3−_ *_x_*Br*_x_* perovskite QDs with excitation peak at 646 nm were synthesized as Joseph M. Luther's reported method with some modification.^39^ PbBr_2_ (0.03 g) and PbI_2_ (0.1 g) mixtures along with 10 mL of octadecene (ODE) were loaded into a 50 mL three‐neck flask, and degassed at 120 °C for 30 min under vacuum. The oleylamine and oleic acid (OAm and OA, 0.5 mL each) acted as ligands and were injected into the flask at 120 °C under protection of N_2_ after being preheated to 70 °C. Then the flask was put in vacuum again until the mixtures completely dissolved and no gas released. After that, the Cs‐oleate (1 mL, 0.1 m in ODE), prepared by dissolving CsCO_3_ in ODE and OA at 150 °C, was quickly injected at 170 °C. After 5 s, the reaction mixture was quenched by immediately immersing it in ice bath, and the color turned to dark red. Finally, the colloidal QDs were precipitated by tert‐butanol and separated via repeatedly centrifugation.

*Device Fabrication*: The as‐grown MoS_2_ films were transferred on Si/SiO_2_ substrate by PMMA‐assisted method, that is, first spin‐coated a PMMA supporting layer on MoS_2_ films, then lifted off the PMMA/MoS_2_ layers by NH_4_OH solution (25%) and followed by removing the PMMA layer in acetone. Then Au (100 nm) source and drain contacts with a 2 µm in length and 20 µm in width channel, which was defined by standard photolithography and electron‐beam lithography, were fabricated on MoS_2_ layer by thermal evaporation. The MoS_2_ samples were annealed at ≈250 °C for 2 h under Ar/H_2_ (100/10) to remove resist residue and improve contact conductance as previously reported.^40^ After that, the synthesized perovskite QD diluted solution (0.25 mg mL^−1^) was uniformly spin‐coated onto MoS_2_ layer with a speed of 2000 rpm. The surface ligands' density was controlled by ethyl acetate and 1‐octane (3:1,v/v) to increase conductance before spin‐coating. Finally, the device was annealed at 60 °C for 15 min to evaporate the organic solvent and improve contacts at the interface for electrical measurements.

*Optical and Electrical Measurements*: The TEM images of the perovskite QDs were taken on an FEI‐Tecnai G2 F20 TEM instrument with an accelerating voltage of 200 keV. Raman and PL spectra were measured by a confocal Raman microscopic system (Horiba Jobin Yvon HR800) with a 532 nm laser as the excitation source; the absorption spectra were obtained by UV–visible–NIR spectrometer (Cary 5000). The KPFM measurement was performed on a Bruker Dimension Icon with a Pt/Ir‐coated probe (SCM‐PIT, *K* = 2.8 N m^−1^, Bruker) by amplitude‐modulated‐KPFM mode at ambient atmosphere. Before measuring, the work function of tip was calibrated by Cu foil, which work function is about 4.8 eV. UPS measurements were performed using He I (21.22 eV) excitation lines as excitation sources in an ultrahigh vacuum (2E^−8^ mbar) chamber (Thermo Scientific ESCALab 250Xi). A confocal two‐photon fluorescence lifetime image microscope system (ARsiMP‐LSM‐Kit‐Legend Elite‐USX) was employed for the FLIM and corresponding TRPL measurements. The electrical measurements were characterized by a semiconductor parameter analyzer (Keithley 4200‐SCS) combined with a probe station at room temperature, and a 532 nm diode laser (spot size ≈1 mm) with a tunable attenuator was used as the illumination source.

## Conflict of Interest

The authors declare no conflict of interest.

## Supporting information

Supplementary
