# Supplementary material for: All‐Inorganic Perovskite Quantum Dot‐Monolayer MoS2 Mixed‐Dimensional van der Waals Heterostructure for Ultrasensitive Photodetector
Source: Adv Sci (Weinh). 2018 Sep 21;5(12):1801219. doi: 10.1002/advs.201801219 (PMC6299711; doi:10.1002/advs.201801219)
Supplement: Supplementary file 1 — Supplementary [file ADVS-5-1801219-s001.pdf]

Copyright WILEY-VCH Verlag GmbH & Co. KGaA, 69469 Weinheim, Germany, 2018.

ADVANCED SCIENCE
Open Access

Supporting Information

for *Adv. Sci.,* DOI: 10.1002/advs.201801219

All-Inorganic Perovskite Quantum Dot-Monolayer MoS_2 Mixed-Dimensional van der Waals Heterostructure for Ultrasensitive Photodetector

Hualin Wu, Haonan Si, Zihan Zhang, Zhuo Kang, Pingwei Wu, Lixin Zhou, Suicai Zhang, Zheng Zhang, Qingliang Liao, and Yue Zhang**

Copyright WILEY-VCH Verlag GmbH & Co. KGaA, 69469 Weinheim, Germany, 2018.

Supporting Information

All-inorganic perovskite quantum dot-monolayer MoS_2 mixed dimensional van der Waals heterostructure for ultrasensitive photodetector

Hualin Wu, Haonan Si, Zihan Zhang, Zhuo Kang, Pingwei Wu, Lixin Zhou, Suicai Zhang, Zheng Zhang, Qingliang Liao, Yue Zhang**

H. Wu, Dr. H. Si, Z. Zhang, Dr. Z. Kang, P. Wu, L. Zhou, S. Zhang, Dr. Z. Zhang, Prof. Q. Liao and Prof. Y. Zhang
State Key Laboratory for Advanced Metals and Materials, School of Materials Science and Engineering, University of Science and Technology Beijing, Beijing 100083, P. R. China.

Prof. Y. Zhang
Beijing Municipal Key Laboratory of New Energy Materials and Technologies, University of Science and Technology Beijing, Beijing 100083, P. R. China.

*E-mail: zhuokang@ustb.edu.cn (Z. Kang), yuezhang@ustb.edu.cn (Y. Zhang)

S1. Photoluminescence spectra.

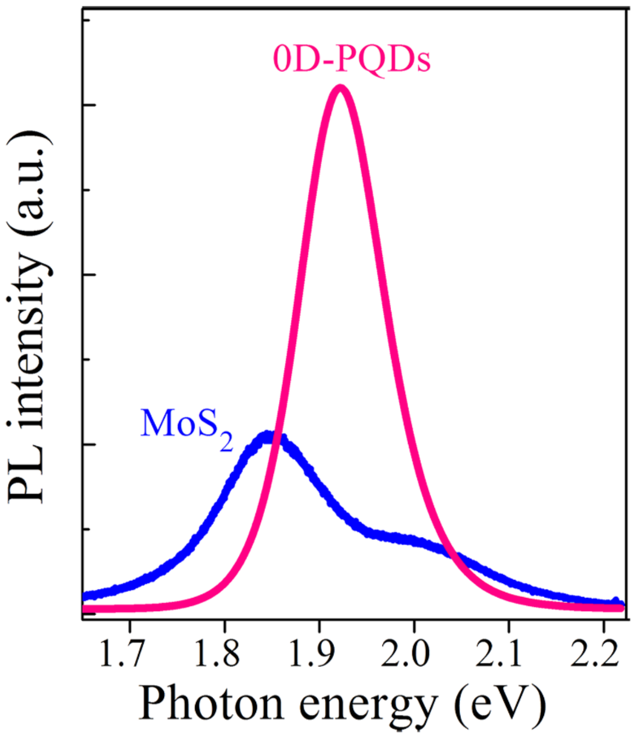

Figure S1. Photoluminescence (PL) spectra of 0D-PQDs and monolayer MoS_2.

S2. Current on-off ratio of the PQDs/MoS_2 MvdWH based phototransistor.

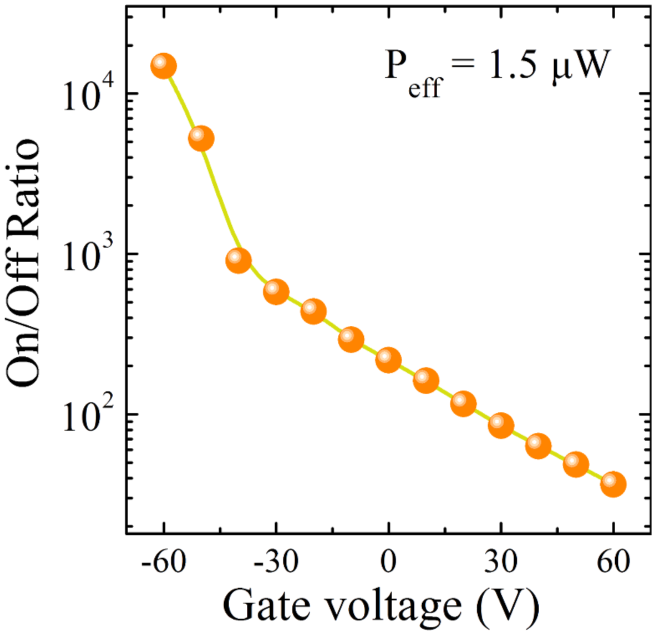

Figure S2. Current on-off ratio of the PQDs/MoS_2 MvdWH based phototransistor under 532 nm, 1.5 µW illumination power.

S3. Output characteristics of the PQDs/MoS_2 MvdWH based phototransistor.

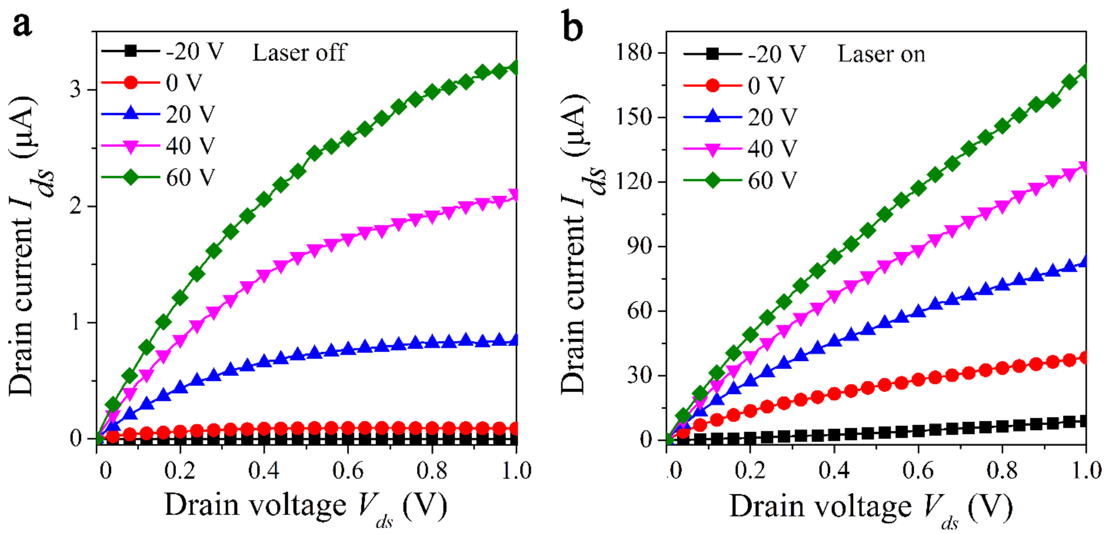

Figure S3. Output characteristics of the PQDs/MoS_2 MvdWH based phototransistor under a) dark and b) illumination (λ = 532 nm and P = 1.5 μW).

S4. Photogating effect modulated by the applied gate voltage.

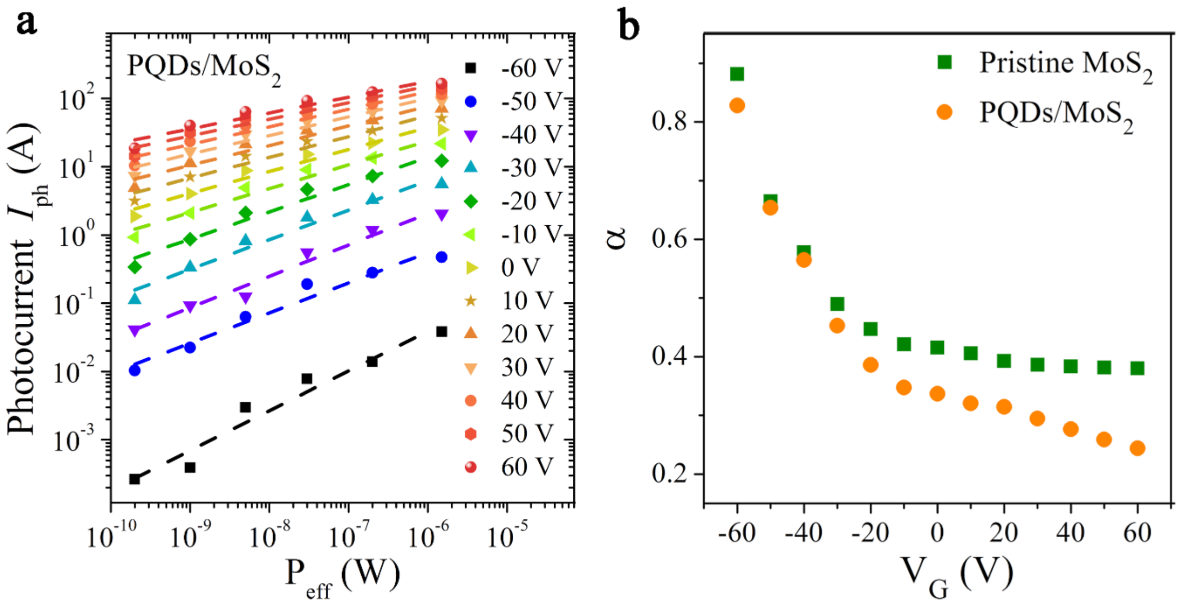

Figure S4. Photogating mechanism modulated by the applied gate voltage. a) Photocurrent versus efficient laser power at different back-gate voltages. b) Exponent α at each gate voltage for the PQDs/MoS_2 and pristine MoS_2 devices, respectively.
